# Verbal and physical violence towards hospital- and community-based physicians in the Negev: an observational study

**DOI:** 10.1186/1472-6963-5-54

**Published:** 2005-08-15

**Authors:** Tal Carmi-Iluz, Roni Peleg, Tami Freud, Pesach Shvartzman

**Affiliations:** 1 The Department of the Family Medicine, Faculty of Health Sciences, Ben-Gurion University of the Negev, Beer-Sheva, Israel; 2 Sial Research Center for Family Medicine and Primary Care, Faculty of Health Sciences, Ben-Gurion University of the Negev, Beer-Sheva, Israel

## Abstract

**Background:**

Over recent years there has been an increasing prevalence of verbal and physical violence in Israel, including in the work place. Physicians are exposed to violence in hospitals and in the community. The objective was to characterize acts of verbal and physical violence towards hospital- and community-based physicians.

**Methods:**

A convenience sample of physicians working in the hospital and community completed an anonymous questionnaire about their experience with violence. Data collection took place between November 2001 and July 2002. One hundred seventy seven physicians participated in the study, 95 from the hospital and 82 from community clinics. The community sample included general physicians, pediatricians, specialists and residents.

**Results:**

Ninety-nine physicians (56%) reported at least one act of verbal violence and 16 physicians (9%) reported exposure to at least one act of physical violence during the previous year. Fifty-one hospital physicians (53.7%) were exposed to verbal violence and 9 (9.5%) to physical violence. Forty-eight community physicians (58.5%) were exposed to verbal violence and 7 (8.5%) to physical violence. Seventeen community physicians (36.2%) compared to eleven hospital physicians (17.2%) said that the violence had a negative impact on their family and on their quality of life (p < 0.05). The most common causes of violence were long waiting time (46.2%), dissatisfaction with treatment (15.4%), and disagreement with the physician (10.3%).

**Conclusion:**

Verbal and/or physical violence against physicians is common in both the hospital and in community clinics. The impatience that accompanies waiting times may have a cultural element. Shortening waiting times and providing more information to patients and families could reduce the rate of violence, but a cultural change may also be required.

## Background

Over recent years Israel, as well as other countries, has witnessed an increase in the prevalence of acts of violence. This rise is seen in workplaces, in recreation sites, on the roads, within the family and even in schools. The mass media are full of reports on violent acts. Violence does not necessarily involve physical contact; it can be verbal or mental. Sometimes psychological or verbal abuse has more severe consequences than acts of physical violence.

Health service providers in hospitals and community clinics are often exposed to verbal and even physical violence that can engender frustration and despair [1-10]. Violent acts against workers have been defined as "any event that the worker is threatened or attacked by another person due to his job" [3,9]. Many physicians feel threatened by verbal and physical violence at work [4,9]. Physicians in emergency medicine, psychiatrists and primary care physicians are at increased risk of violent acts from patients and families [3,4,10].

Studies from England from 1989, 1991, and 1997 have shown that verbal abuse is the most frequent type of violence reported by British physicians (25–91%) [5-7] compared to physical violence (1–11%). However the latter has significantly affected those physicians who were exposed to it leading in some cases to depression, insomnia, post-traumatic stress disorder, agoraphobia [4] and even a level of fear and/or anxiety that can cause work absenteeism [8]

In the US the rate of violence is even higher. Between the years 1980–1990, 106 healthcare workers died as a result of violence: 27 pharmacists, 26 physicians, and 53 nurses [2]. Another survey of 170 university hospitals in the US showed that 57% of all emergency room employees had been threatened by weapons over the five-year period prior to the survey [10].

Seventy percent of the physicians and 90% of the support staff working in a hospital emergency room in Israel reported violent acts, mostly verbal abuse [1].

The main reasons for these outbursts were long waiting times, dissatisfaction with treatment, something that was said that the patient took exception to, and in some cases the influence of alcohol and/or drugs on the perpetrator of the violence [1]. No other studies of this type have been reported from Israel.

The aim of the present study was to assess violence against physicians in the southern Negev region of Israel, and to compare rates in the hospital with those in community clinics.

## Methods

### Setting

The study was conducted within the framework of the Southern District of Clalit Health Services, Israel's largest HMO that serves about 60% of the population. The population of the Negev region in southern Israel numbers about 530,000, most of a low to middle socioeconomic level. The largest city in the area is Beer-Sheva with about 200, 000 residents. The Soroka University Medical Center is located in Beer-Sheva. The rest of the Negev's residents live in smaller communities.

The study was conducted among 95 physicians in all major departments of the Soroka Medical Center (internal medicine, surgery and pediatrics) and 82 family physicians and pediatricians working in primary care clinics of the Clalit Health Services in the Negev. Hospital physicians were sampled based on the physician roster of the Soroka University Medical Center and the community-based physicians based on the physician roster of the Southern District of the Clalit Health Services. In the few cases in which the physicians stated that they work both at the hospital and in the community, their primary place of work was used for statistical analyses.

### The study instrument

All participants completed an anonymous questionnaire, consisting of 36 items, on their experiences with and attitudes towards violence. The questionnaire included demographic and personal data, reports on exposure to verbal and physical abuse over the previous year, information about how they dealt with the violence and their attitude to it. Most of the items were multiple-choice questions, with one possible answer, but in a small number of questions we used an open format. An example of a closed question is: How did you react to an episode of verbal violence? The response options were: 1) I ignored it; 2) I made a verbal response; 3) I called for security personnel; 4) I called the police; 5) I lodged a complaint with the police; 6) other (with space to write a detailed response).

We developed the questionnaire specifically for the study after a thorough review of the literature on the subject. The questionnaire was revised in light of the results of a pilot study of 15 physicians who did not participate in the study itself. In the explanation that preceded the questionnaire we stated that the term "verbal violence" could be defined as an attempt to attack another person by means of harsh words, cursing, an aggressive manner of speech, threats, or any other manner of speech that it is not acceptable, but does not lead to physical injury. "Physical violence" could be defined as any form of attack that has a physical component.

Data were entered into the Epi-Info 6 program and were analyzed using the SPSS statistical package. T-tests and χ^2 ^tests were used as appropriate. Statistical significance was set at P < 0.05.

## Results

The study population was comprised of 177 physicians from a roster of 200 (105 hospital-based and 85 community-based physicians), so the response rate was 88.5%. Ninety five (53.7%) worked in the Soroka Medical Center and 82 (46.3%) worked in the community. Sixty nine (39.7%) were women. The mean age was 41 ± 8.6 years. Eighty physicians (45.5%) were Israeli born and 52 (29.5%) were from the former USSR. The socio-demographic characteristics of the participants are presented in Table 1.

**Table 1:** Comparison of study groups for sociodemographic and professional data (n = 177; some variables have missing values).

**Variable**	**Hospital physicians**	**Community physicians**	**All**	**P**
Mean age (years)	40.0 ± 9.4	42.3 ± 7.3	41.0 ± 8.6	NS

Gender [N (%)]				
Female	28 (30.1)	41 (50.6)	69 (39.7)	<0.01
Male	65 (69.9)	40 (49.4)	105 (60.3)	

Country of birth [N (%)]				
Israel	53 (56.4)	27 (32.9)	80 (45.5)	<0.05
East Europe	9 (9.6)	12 (14.6)	21 (11.9)	
West Europe	2 (2.1)	0	2 (1.1)	
Former USSR	22 (23.4)	30 (36.6)	52 (29.5)	
Other	8 (8.5)	13 (15.9)	21 (12.0)	

Professional status [N (%)]				
Specialist	44 (46.3)	56 (69.1)	100 (56.8)	<0.01
Non-specialist	51 (53.7)	25 (30.9)	76 (43.2)	

Mean professional experience (years)	12.3 ± 9.7	14.2 ± 7.3		NS

### Verbal violence

Ninety-nine of the 177 participating physicians (56.0%) stated that during the previous year they had been exposed to at least one case of verbal abuse. Fifty-one hospital physicians (53.7%) were exposed to verbal abuse by patients and 69 (72.6%) by family members, compared to 48 (58.5%) and 40 (49.4%), respectively for community-based physicians (p < 0.01 for verbal violence by family members).

Fifty six physicians (73%) reported having experienced 1–3 cases of verbal abuse by their patients over the past year (mean 4.3 ± 6.8). Seventy eight physicians indicated the number of times they had been exposed to verbal violence from family members of patients. Of these, 57 physicians (73%) mentioned that during the last year they had experienced 1–4 cases (mean 3.65 ± 3.39).

### Physical violence

Sixteen of the 177 physicians (9%) were exposed to physical violence over the previous year. Nine doctors (5.1%) experienced acts of physical violence by their patients, and 7 doctors (4%) by family members of their patients. Three hospital physicians (3.2%) were exposed to physical violence by patients and 6 (6.3%) by family members, compared to 6 (7.3%) and 1 (1.2%), respectively for community-based physicians.

Nine physicians (5.1%) were punched, 3 (1.7%) had an object thrown at them, 2 (1.1%) were held with excessive pressure, and 1 (0.6%) was punched and had an object thrown at him.

### The site of violence

#### Verbal violence

Forty three of 113 respondents (38.1%) said that the act of verbal violence took place on the ward, 33 (29.2%) in the physician's office, and 29 (25.7%) in the emergency room.

#### Physical violence

Five respondents (2.8%) said that the act of physical violence took place on the ward, 5 (2.8%) in the physician's office, and 5 (2.8%) in the emergency room.

### Physicians' perceptions of the degree of risk from violence

Twenty two of 170 respondents (13%) said that they felt that their health had been endangered as a result of verbal violence the past year, 18 (10.6%) to a minor degree and 2 (1.2%) to a major degree. Thirty nine of 174 respondents (22.4%) said that they felt that their life had been endangered as a result of physical violence the past year.

### The impact of violence on physicians (Table 2)

**Table 2:** The impact of violence on physicians' lives.

**Variable**	**Hospital physicians N (%)**	**Community physicians N (%)**	**P**
Did violence affect life outside of work?			
No	53 (82.8)	30 (63.8)	<0.05
Yes	11 (17.2)	17 (36.2)	

Length of time violence had this effect?			
One day	43 (71.7)	31 (67.4)	NS
Up to one month	12 (20.0)	9 (19.6)	
More than one month	5 (8.3)	6 (13.0)	

One hundred eleven physicians answered a question about the effect of violence on their family life and their overall quality of life. Twenty eight (25.5%) stated that violence had a detrimental effect on their lives. Seventeen of those who said that violence had a detrimental effect on their lives (60.7%) were females compared to 11 males (39.3%) (p < 0.05). Eleven hospital-based physicians (17.2%) and 17 community-based physicians (36.2%) said that violence had a detrimental effect on their life (p < 0.05).

Twenty one physicians (19.8% of 106 who answered the question) said that the detrimental effect lasted for up to one month, 11 (10.4%) said the effect lasted over one month.

### The perceived causes of violence

The most frequent causes of violent acts cited by the physicians were: long waiting periods (46.2%), patients' dissatisfaction with the treatment (15.4%), patients' disagreement with the physician (10.3%), no perceivable reason (9.4%), patients' unjustified request for a medical certificate (4.2%), other reasons (14.5%). Thirty two community-based physicians (50%) stated that the reason for violence was long waiting times in their clinic.

### Handling of the incident

Seventy three of the 106 physicians (68.9%) who answered this question said that acts of violence weren't treated in any way by the hospital or the community clinic, 19 (17.9%) said that the security staff sent the attacker away, and 10 (9.4%) said that the act was treated through legal channels.

### Physicians' satisfaction with the manner in which the authorities handled the act

Of 100 physicians who answered this question, 43 weren't satisfied with the way the incident was handled, 32 were satisfied, and 25 were partially satisfied.

### Why did physicians not file a complaint with the local police?

Of 94 physicians who answered this question, 43 (45.7%) felt that the incident did not justify a complaint to the police, 17 (18.1%) were satisfied with the attacker's apology, and 15 (16%) said that they were not prepared to go to court over the incident.

### Physicians' attitudes to violence

Sixty percent of the participating physicians, including those who were not personally exposed to acts of violence over the previous year, believed that violence represents a serious threat. Only 6 (3.4%) thought that violence is not a serious problem for physicians today. Eighty three percent stated that they were not trained to prevent or deal with acts of violence.

## Discussion

The deterioration of the economic and security situation has led to increasing violence in Israel. Hospital- and community-based physicians, who care for patients affected by physical and mental distress, may be exposed to violent acts at the workplace. Inadequate working conditions, with a small number of physicians caring for a large numbers of patients, overload the health care system causing prolonging waiting times that may trigger violent outbursts on the part of frustrated patients and their families. According to Israeli Ministry of Health data there were 1440 cases of violence in Israeli hospitals in 2001 compared with 675 in 2000. Of these, 432 were physical violence, 739 were verbal violence and 269 involved loss of property. The police intervened in 285 cases of violence in some cases making arrests and filing charges [11].

The aim of this study was to characterize verbal and physical violence against physicians in hospital and in community clinics in the Negev as reported by the physicians themselves and to assess their attitudes to these acts. Ninety nine of 177 participating physicians (56%) reported at least one incidence of verbal violence over the previous year. Sixteen of 177 (9%) reported at least one case of physical violence over the same time period. The response rate of 88.5% is high and was achieved by virtue of a data collection system that included a personal appointment with each physician, use of a relatively short questionnaire that had as its heading the official logo of the Department of Family Medicine, and the fact that the study population was highly motivated and inclined to participate in a study on an issue that is close to their hearts and important to them [12].

In terms of the degree of physical and verbal violence, our findings are similar to those reported from Great Britain [5-7], which show that verbal abuse is the most frequent type of violence, but are better than those reported from a primary care department in a Kuwaiti hospital in 1999 [13]. There, 86% of the physicians surveyed reported being exposed to verbal violence, and 28% to physical violence, with severe injury or even death in 7% of the cases. In the USA 106 health employees died as a result of violence between the years 1980–1990 [2].

In a study conducted in the Barzilai hospital in Ashkelon, Israel 70% of the participating physicians reported acts of verbal violence and 38.5% of the emergency medicine personnel experienced physical violence [1]. It is possible that work in an emergency room entails greater risks of violence than other hospital departments.

Significantly more hospital-based physicians (72.6%) experienced verbal violence by family members than community-based physicians (49.4%). Possible explanations for this finding include:

1. Hospital physicians are exposed more to confrontations with patients' family members who visit their hospitalized relatives and often want to be involved in decisions relating to them, while most patients come to community clinics alone.

2. Family members may feel less constrained to confront hospital physicians whom they don't know than family physicians with whom they may have an ongoing relationship.

Studies from around the world show that acts of violence have a negative effect on the physician's family life and quality of life. In the study conducted in Kuwait 86% of the physicians who experienced violence reported that it caused insomnia, depressions and other effects [14]. In the present study we found that 36.2% of the community-based physicians reported a negative impact on their family life and quality of life, compared to 17.2% of the hospital-based physicians. This may be explained by the long-term professional relationship that the family physician has with the violent patient, compared to the short-term contact that the hospital physician has with the patient.

The most common triggers for violent acts were long waiting time, dissatisfaction with the treatment and/or disagreement with the physician. Similar findings were reported from the study conducted in Barzilai hospital [1]. It is possible that the impatience that accompanies a long waiting time and acts as a trigger for violence is affected by cultural norms as well.

In two thirds of the cases the incident was not handled by the hospital or the community clinic, perhaps because the involved physician may not have perceived it as severe, although even the severe cases were not always handled by the hospital or the police. Some physicians may hesitate to report acts of violence because they do not expect the medical administration to come to their aid. It is also possible that physicians' cultural background may influence their reactions and approach to handling the incident. Most participating physicians, including those who did not experience acts of violence over the previous year, thought that violence is a significant issue for physicians. In Kuwait the overwhelming majority of study physicians said they were concerned about violence in the workplace and felt that physicians should receive formal instruction on how to handle these situations [14]. We also found that most of the physicians felt that they were not trained to prevent or cope with the violence at the workplace, indicating that even though violence exists and is considered a serious problem by physicians, it does not appear to be a major issue for the medical establishment and there are no serious attempts to minimize the phenomenon.

There are some limitations to our study. It was undertaken in major hospital departments, but did not include the emergency department where tension and violence may appear. This may be a reason for the relatively low rate of violence in the hospital. On the other hand it should be noted that many of the physicians who were interviewed as ward physicians actually spend part of their work time in the emergency room. Another limitation is the lack of information regarding the violent patient or his/her family, i.e., do they have a personality disorder or psychiatric problems, or perhaps just a normal person who felt that the waiting time was unreasonable.

The issue of triggers of violence was not dealt with in a way that fully recognizes the complexity of attributions made by people involved in the violent act, since no attempt was made to corroborate the attributes by interviewing the assailants. Although this type of expanded study might have contributed to the discussion of possible triggers, we feel that the methodology used, despite this limitation, is valid.

The results of this study add to our current knowledge because of its comparison of physical and verbal violence on the part of patients and/or their family, and the comparison between hospital- or community-based physicians from the same geographic area.

In order to start to alleviate the problem of violence towards physicians steps have to be taken to address the triggers listed above. Waiting times have to be shortened. This can be accomplished by better planning of medical education and manpower, and by increasing the number of physicians in the clinics to improve the patient-physician ratio. It is also important to change cultural norms that may serve as triggers to violence, but this strategy requires a long period of time and would require further studies before its implementation. In the short term it is important to organize workshops to train physicians to prevent and deal with violent incidents. In these training sessions physicians will be taught to be patient, to provide appropriate and relevant information, and to show respect towards patients and family members. At the same time hospital and clinical security should be increased and enforced; the strict rules for treating violent people should be designed and implemented.

## Declaration of competing interests

The author(s) declare that they have no competing interests.
